# On the blind spot: ethical conflicts of health care professionals in addressing opioid diversion - a template study protocol for multinational qualitative research

**DOI:** 10.1186/s12910-026-01581-6

**Published:** 2026-08-28

**Authors:** Annette Binder, Sonja Mathes

**Affiliations:** 1 Department of General Psychiatry and Psychotherapy, Addiction Medicine and Addiction Research Section, University Hospital Tübingen, Tübingen, 72076 Germany; 2 German Center for Mental Health (DZPG), Partner Site Tübingen, Tübingen, 72076 Germany; 3 Department of Dermatology and Allergology, School of Medicine and Health, Technical University of Munich, Munich, 80802 Germany; 4 Institute for History, Theory, and Ethics of Medicine, Johannes Gutenberg University Mainz, Mainz, 55122 Germany

**Keywords:** Opioid diversion, Health care professionals (HCPs), Ethical dilemmas, Qualitative research, Moral distress, Substance use disorder (SUD), Reflexive thematic analysis, Professional ethics in medicine, Opioid-related harm reduction

## Abstract

**Background:**

Opioid diversion—the unauthorized redirection of prescription opioids—is a growing global concern with significant public health implications. While diversion can occur at many points in the supply chain, instances involving healthcare professionals (HCPs) are particularly complex, as they often occur within clinical settings and most often involve self-medication, substance use disorders among healthcare professionals, or peer dynamics. These cases raise not only legal and professional concerns but also profound ethical dilemmas. To date, the ethical perspectives of HCPs in relation to opioid diversion have been underexplored. This multinational qualitative study aims to investigate how HCPs experience and navigate ethical conflicts related to opioid diversion in clinical contexts. The study focuses on the lived experiences and moral reasoning of HCPs who encounter diversion, either among colleagues or in relation to their own practice. It seeks to explore and identify barriers to seeking help and accessing harm reduction, and to understand how these challenges vary across different healthcare systems.

**Methods:**

Semi-structured interviews will be conducted with HCPs working in clinical settings where opioids are used. A fictional scenario involving suspected diversion among peers will be used to encourage reflective discussion. This approach is intended to minimize social desirability bias and potential legal concerns in comparison to assessing experienced diversion. The interview guide is structured around the five steps of ethical analysis as outlined by Marckmann, supporting a theory-informed exploration of individual and systemic dilemmas. Data will be analyzed using reflexive thematic analysis (RTA), allowing for nuanced insights into both explicit reasoning and latent moral constructs. Recruitment of HCPs takes place in hospital departments characterized by frequent opioid use. The study protocol is intended for international implementation to support cross-national comparative analysis.

**Discussion:**

This study will generate a deeper understanding of the ethical tensions HCPs face in the context of opioid diversion. By identifying structural and cultural drivers behind moral distress, the findings may help address stigma and inform the development of ethically guided harm reduction strategies as well as supportive structures for HCPs. The cross-national comparative perspective aims to contribute to an international framework for reducing opioid-related harm in healthcare.

**Supplementary Information:**

The online version contains supplementary material available at https://doi.org/10.1186/s12910-026-01581-6.

## Background

The global opioid crisis continues to pose significant public health challenges, including the expansion of illegal markets, the distribution of counterfeit medications, and the misuse of prescription drugs. While these issues have received increasing attention from researchers and policymakers, one critical aspect remains largely overlooked: the “loss” of opioids within healthcare systems. This phenomenon of unlawful redirection of prescription medications from legitimate medical channels into illicit use is referred to as “diversion”. It can occur at virtually any point in the pharmaceutical supply chain, from manufacturing and distribution to prescribing and dispensing, all the way to the patient. Previous research has primarily focused on the motivations of patients or family caregivers who diverted prescribed opioids, for example, those used in substitution therapy [1], pain management [2, 3] or hospice care [4]. However, it has been known for many years that healthcare professionals (HCPs) themselves can also engage in diversion within the healthcare system [5]. Unlike diversion through theft or street-level trafficking, cases involving HCPs often occur within the boundaries of clinical environments and often include personal use or self-medication. There is reason to believe that at least some HCPs involved may be experiencing an opioid use disorder (OUD). This form of diversion is not only professionally and legally problematic, but also morally distressing for all individuals involved. Due to the complexity of healthcare environments characterized by continuous interactions between patients, healthcare professionals (such as nurses, physicians, pharmacists and technicians) among each other, technologies, and intricate organizational workflows [6].

Therefore, opioid diversion can profoundly impact numerous stakeholders—physically, psychologically, and morally. It introduces challenging questions regarding responsibility, vulnerability, and the inherent pressures of medical practice. Divergent moral perspectives in situations where diversion is suspected - or witnessed - can lead to ethical dilemmas - that is a conflict of contradicting moral concerns, necessarily resulting in violation of one of the positions [7]. Due to the highly sensitive social and legal frameworks surrounding the topic of Diversion in Healthcare settings, ethical dilemmas are difficult to resolve.

### Research goals and objectives

While diversion can involve a variety of drug classes—such as stimulants, sedatives, and anxiolytics—opioid diversion has gained particular relevance due to the ongoing opioid crisis, which is most severe in North America but increasingly recognized as a global public health concern. Therefore, this study specifically focuses on opioids. With this study, we aim to contribute to the understanding of the complex moral issues surrounding the diversion of opioids by healthcare professionals — as witnessed or suspected by their colleagues — and to explore the limitations to openly discussing such perspectives within their respective healthcare settings. To this end, his study will focus on three key objectives:

First, this study seeks to gain deeper insights into how HCPs perceive opioid diversion in clinical practice, especially when it occurs among colleagues and potentially involves their own use. Particular attention will be given to the ethical dilemmas and mechanisms of stigmatization they encounter in such situations. By conducting qualitative interviews, the study aims to initiate reflective and detailed conversations with HCPs working in clinical settings. These conversations will be designed to carefully elicit lived experiences as well as moral reasoning in ethically complex scenarios. At this, we firstly want to shed light on how participants make sense of their roles and responsibilities in opioid stewardship. Drawing on the perspectives of professionals who have either passively witnessed or been actively involved in cases of diversion, the study will help define the structural and cultural conditions that facilitate or hinder ethical decision-making.

Secondly, we identify key ethical challenges experienced by HCPs in relation to opioid diversion. Doing so will contribute to a more nuanced understanding of professional vulnerability and support the development of interventions that prioritize both patient safety and caregiver well-being.

Thirdly, this study aims to contextualize opioid diversion within a broader public health framework by examining how individual cases and ethical decisions in clinical practice are linked to systemic consequences. By highlighting the interplay between personal responsibility and institutional structures, we seek to understand how opioid diversion by HCPs may affect public trust, prescribing cultures, and the overall effectiveness of opioid stewardship programs. In doing so, the study contributes to a more integrated perspective on how micro-level ethical conflicts relate to macro-level health outcomes—such as the prevention of substance misuse, the protection of vulnerable populations, and the promotion of ethical clinical environments. These insights are essential for developing sustainable, ethically grounded public health strategies that address the root causes of diversion without reinforcing stigma or punitive surveillance cultures.

The findings of this study aim to inform the development of future strategies focused on preventing opioid diversion, strengthening peer support, and enhancing ethical training, thereby contributing to efforts to mitigate the opioid crisis.

Our objectives will be guided by the following research questions:

1. How do HCPs perceive and interpret unauthorized opioid diversion in their clinical environments?
2. What are the ethical dilemmas that arise for HCPs who encounter opioid diversion?
3. What barriers to harm reduction emerge as a result, particularly in terms of supporting HCPs with OUD?
4. How does opioid diversion by HCPs impact public health goals, including trust in healthcare systems, prescribing practices, and institutional responses to substance use?
5. How do ethical dilemmas of diversion in HCP vary globally and what are the differences at barriers to harm reduction across different health care systems?

## Methods

Given the study’s exploratory nature, which seeks to explore lived experiences, ethical reasoning, and context-specific challenges related to opioid diversion, a qualitative research approach is particularly appropriate. Qualitative methods enable an in-depth understanding of how HCPs interpret complex situations, navigate moral tensions, and construct meaning within their professional roles. Through semi-structured interviews and reflexive thematic analysis (RTA) [8], the study will generate nuanced insights into ethical challenges that are difficult to capture through quantitative approaches.

To ensure methodological rigor and transparency in the design and reporting of this qualitative study, we followed the Consolidated Criteria for Reporting Qualitative Research (COREQ) checklist [9] throughout the planning and implementation phases. The COREQ checklist provided a structured framework to guide the development of the study design, data collection, and analysis procedures, thereby supporting quality assurance. For the reporting of findings, we additionally adhere to the Reflexive Thematic Analysis Reporting Guidelines (RTARG) [10], which align with our use of reflexive thematic analysis and provide detailed criteria for presenting and contextualizing the results in a coherent and reflexive manner.

### Ethics

Participants will receive detailed study information, including data protection measures. The purpose of the cross-national analysis will also be explained. Along with the study information, participants will be provided with a consent form and a demographic survey, which they are required to complete and return to the study team. The demographic survey collects data on gender, age, professional group, and relevant aspects of their work context. Participants will give their informed consent in writing by signing the consent form and will also provide verbal consent prior to the start of the interview.

For the planned national individual studies, it is noted in the local ethics applications and in the participant information that the collected (anonymized) data will be used for a cross-national analysis extending beyond each individual research project.

### Theoretical framework

Ethics, within the field of medical ethics, establishes methods that serve to conduct comprehensive and systematic reflections in medical and healthcare contexts, and to arrive at an overall ethical judgment in complex situations. An ethical analysis provides a framework for such complex decision-making. A comprehensive, structured approach to such an ethical analysis has been suggested by Marckmann [11].

Marckmann’s advocacy for the fundamental steps of ethical analysis can be understood as a widely applicable template for an ethical analysis [11]. In this study it serves as the guiding framework for developing an interview guide. Marckmann outlines six steps of a sound ethical analysis, which include:

1. A precise description of the initial situation, including all (theoretical) courses of action;
2. Definition of the normative criteria for choosing between these options;
3. Evaluation of the available options based on the established normative criteria;
4. Synthesis of the individual assessments into an overall judgment, including appropriate weighting;
5. Derivation of practical recommendations for action in the given situation;

Marckmann then adds:

6. an evaluation of the proposed courses of action.

For our analysis, participants are to be guided (hermeneutically) through the first five steps of ethical analysis according to Marckmann. The last step will not be part of our analysis, as an evaluation is not part of the study. It might however be the case that participants describe their own lived experiences, including the follow up of actions they might have taken, thereby covering the sixth point as well.

This approach is chosen because it provides structure while allowing participants the flexibility for individual approaches. For our setting, two key guiding questions are central: (1) The identification and contextualization of personal normative views and existing dilemmas of HCP, and based on this, (2) the characterization of strategies to ethically and effectively address diversion.

### Instruments

Semi-structured interviews will be conducted, using a fictional scenario involving diversion in a clinical setting by a HCP as a stimulus for further discussion. The use of a fictional scenario, as opposed to direct inquiries into personal experiences, facilitates more open participant responses by reducing concerns over legal consequences and avoiding the need to disclose potentially morally sensitive behavior, such as choosing not to report suspected cases. The interview guide functions as a tool to guide participants step by step through the reflective stages of ethical analysis in a structured and purposeful manner. The scenario and the interview guide were developed by the authors. During the development process, both instruments were presented in the interdisciplinary “Research Workshop for Qualitative Methods” organized by the Coordination Office for Health Services Research, a joint initiative of the Institute of Occupational and Social Medicine and Health Services Research and the Institute of General Practice and Interprofessional Care at the University Hospital Tübingen. Based on feedback from the interdisciplinary research group, composed of experts in health services research, the materials were revised accordingly.

### Fictional scenario

The fictional entry scenario was developed through an iterative process involving researchers from various disciplines—such as nursing and medicine—as well as from different medical specialties. Interdisciplinary and interprofessional input was integrated through the framework of the “Research Workshop for Qualitative Methods”. The initial draft of the scenario was developed by the two authors and refined through discourse and feedback generated within the workshop setting.

The following criteria were considered important for the final version of the scenario:

- Setting: Neutral, to ensure high transferability to the participants’ own professional contexts.
- Element suggestive of potential diversion: various examples were provided to ensure high transferability.
- Portrayal of the person suspected of diversion: Neutral or positive. Indications of the presence of OUD are not initially indicated, but will be introduced later during the interview (if the participants do not express this as a reason/assumption).

The final scenario reads as follows:

Imagine the following situation occurs in your current work environment: There is a colleague on your team with whom you have been working for quite some time. This person is well-liked both in the clinic and within the team, and you also hold them in high regard. Lately, however, you have noticed some unusual behavior. For example, whenever opioids need to be administered, this colleague frequently volunteers to hand the medication directly to patients, even when this is not necessarily their responsibility. You may also notice that they tend to recommend or prescribe strong opioids or high dosages more readily when patients report pain. Or perhaps you observe that patients under this person’s care often receive particularly high opioid doses. This leads you to wonder whether this could be a case of diversion.

### Interview guide

The development of the interview guide was informed by ethical analysis, an established approach in medical ethics, following the steps outlined by Marckmann [11]. Table 1 summarizes the interview questions and their alignment with the steps of ethical analysis.

**Table 1:** Interview questions and correspondence to the steps of ethical analysis

Interview Question	Step of Ethical Analysis
1. Please describe how such a situation could realistically unfold in your work environment.	Step 1: Situation
2. What do you think could be the motives behind this person’s diversion?	Step 1: Situation
3. What do you think about this situation and how would you feel in such a situation?	Step 2: Normative Evaluation
4. What do you think about this person?	Step 2: Normative Evaluation
4.1 To what extent could suspicion of diversion change your perception of this person in your working environment?	Step 2: Normative Evaluation
5. What moral aspects do you see as relevant in this conflict?	Step 2: Normative Evaluation
6. What conflicts do you perceive in dealing with this situation?	Step 2: Normative Evaluation
7. What would change in terms of the moral conflicts involved, depending on different motives for Diversion?	Step 2: Normative Evaluation
8. What options for action do you see in this conflict situation?	Step 3: Assessment of Options and Limitations
8.1 Which moral aspects could be considered for each possible solution – and which could not?	Step 3: Assessment of Options and Limitations
8.1 How important are these moral aspects to you in this situation – and why?	Step 4: Synthesis
9. How would you resolve the situation?	Step 4: Synthesis
10. What would be your recommendation in this situation now?	Step 5: Solution

The complete interview guide, including the introduction text, scenario, main interview questions, and probing prompts, can be found in supplementary material 1.

### Sample size

To guide sample size estimation in this qualitative study, we draw on the concept of Information Power as proposed by Malterud, Siersma [12]. Rather than relying on fixed numerical thresholds, this framework posits that the more information a sample holds that is relevant to the study aims, the fewer participants are needed. Five key factors influence this estimation: the aim of the study, sample specificity, use of theory, quality of dialogue, and analysis strategy.

#### Study Aim

The aim of this study is exploratory in nature, focusing on ethical conflicts experienced by HCPs in the context of opioid diversion within the healthcare system. Given the open and interpretative nature of the research question, a larger sample size is warranted to capture the breadth of perspectives.

#### Sample specificity

The sample consists exclusively of HCPs, which offers moderate specificity. While all participants operate within the healthcare system, the diversity of professional backgrounds (e.g., physicians, nurses, pharmacists) and clinical disciplines introduces a degree of heterogeneity, slightly reducing the overall specificity of the sample.

#### Use of established theory

The study employs a theory-informed approach, using ethical analysis frameworks to interpret participant accounts. This theoretical anchoring supports a smaller sample, as the analysis is guided by existing conceptual tools rather than being entirely inductive.

#### Quality of dialogue

The anticipated quality of dialogue is high, as HCPs are generally well-equipped to reflect on and articulate ethical dimensions of their professional practice. This communicative competence enhances the information yield per interview, thus potentially lowering the required sample size.

#### Analysis strategy

The study applies Reflexive Thematic Analysis (RTA) as outlined by Braun and Clarke [8] which facilitates comparative, across-case interpretation. This analytical strategy favors a larger sample to enable meaningful pattern development across diverse participant narratives.

Taken together, these considerations suggest a balanced sample size, large enough to accommodate the exploratory aim and the comparative nature of the analysis, while also benefiting from the theoretical grounding and expected depth of data. Accordingly, the study plans to conduct 15 to 25 interviews with HCPs working in hospital departments where opioids are frequently used, ensuring access to participants with clinical insight and first-hand experience of ethical dilemmas in opioid use and diversion.

### Inclusion and exclusion criteria

The study includes HCPs (e.g., physicians, nurses, etc.) working in hospitals, either in inpatient or outpatient clinical settings. Eligible participants must work in departments where opioids are frequently used, such as anesthesiology, oncology, palliative care, pain medicine, surgery, wound care, infectiology, or addiction medicine (including opioid withdrawal treatment). Individuals working in areas or roles where opioids are not used will not be included.

### Recruitment and sampling

Recruitment will occur through email invitations disseminated via hospital mailing lists, with a targeted focus on departments involved in the administration of opioids. To ensure diversity in institutional contexts, hospitals of varying sizes from different regions of Germany will be specifically targeted during recruitment. The initiation of national research projects worldwide is planned, with these studies currently in the preparatory phase. Recruitment strategies will follow a shared framework, primarily using email invitations via hospital mailing lists and relevant medical institutions. These strategies will be adapted to each country’s healthcare system and institutional structures to ensure feasibility and comparability across sites.

### Data collection

Data will be collected through audio-recorded interviews conducted via videoconference or telephone. Before recording begins, participants will receive a brief review of the study information and be asked to provide verbal consent. All interviews will then be transcribed verbatim.

### Data analysis

Following Braun and Clarke’s [8] approach, Reflexive Thematic Analysis was selected for its capacity to support researchers in actively interpreting patterns of meaning in qualitative data, while offering flexibility to incorporate theoretical frameworks. In line with this study’s aims, RTA will be employed to identify ethically reflective statements prompted by the interview guide, while also allowing for the exploration of latent meanings, including potential stigmatizing attitudes toward individuals with OUD. By centering the researcher’s role and active self-reflection, RTA fosters a careful and deliberate engagement with both the data and the interpretive process [13]. Consequently, RTA encourages researchers to critically and consistently engage with their theoretical and moral assumptions throughout the entire research process [13]. RTA is a structured yet flexible process that encourages researchers to critically reflect on their assumptions throughout the interpretation of qualitative data [8, 13]. The six phases include: (1) familiarisation with the dataset; (2) systematic coding; (3) development of initial themes; (4) reviewing and refining themes; (5) defining and naming themes; and (6) producing the final report.

This analytical process is conducted collaboratively within the research team and complemented at selected stages by the involvement of experts in medical ethics and addiction medicine, in order to ensure intersubjectivity and enhance the rigor of data interpretation. To ensure adequacy—that is, the alignment between the research design and the subject matter—ongoing critical reflection throughout the research process is essential [14]. In cases where the data reveal a substantial presence of narrative elements, it may be appropriate to incorporate or integrate a narrative analysis [15] as an intermediary step within the overall analytic framework.

### International comparative analysis

This study plans to implement the harmonized interview protocol in multiple countries to ensure consistency across sites. After initial qualitative data analysis at the national level, a comprehensive comparative analysis will be undertaken. This multi-step comparative phase will begin with within-region analyses, grouping countries into clusters such as Europe, North America, and other relevant geographical or cultural regions to identify shared and unique thematic patterns within similar sociocultural contexts.

Subsequently, a comprehensive international synthesis will be performed, integrating findings across all participating regions to explore broader cross-cultural similarities and differences. This multi-layered approach will facilitate a nuanced understanding of the data, preserving local contextual richness while enabling systematic comparison at regional and global levels.

Throughout this process, rigorous reflexivity and team collaboration will be maintained to ensure consistency, transparency, and intersubjective validation of interpretations across national and international research teams.

## Discussion

This study protocol offers a unique opportunity to advance progress on reducing opioid-related harm by focusing on a largely neglected yet critical aspect: the ethical conflicts experienced by HCPs in the context of opioid diversion. A nuanced qualitative exploration of these ethical dilemmas can help to uncover underlying barriers that prevent HCPs from addressing issues adequately or seeking support or therapeutic services. By understanding these conflicts for the first time in detail, the study may inform the development of more responsive support systems that acknowledge the moral and emotional complexity faced by HCP in practice.

Moreover, the multinational scope of the study allows for a comparative analysis of how such conflicts are experienced and addressed across different cultural and legal contexts. These insights can contribute to the formulation of a shared framework or action plan to reduce opioid-related harm among HCPs at both regional and global levels.

In the context of diversion, it is essential to proceed with caution in order not to disrupt the sensitive frameworks of healthcare systems around the world: There is a responsibility to address sensitive topics in a manner that does not destabilize individuals or institutions. The impression of courtesy and safety of healthcare systems is crucial for perception of quality of care [16]: Addressing conflicts of these values with diversion could lead to friction in healthcare settings. However, it must also be acknowledged that criminal acts, particularly diversion, pose a significant threat to patient safety, as well as to the wellbeing and safety of healthcare professionals [5]. Therefore, a thoughtful yet critical examination of the issue is not only appropriate but also a moral imperative.

From a practical and operational perspective, several challenges must be anticipated. Recruitment of participants among HCP may prove difficult, as the topic of opioid diversion and associated ethical conflicts is highly sensitive [5]. The prospect of legal consequences for offenders, including conviction and custodial sentences [17], may serve as a deterrent to potential participants [18]. To mitigate these barriers, the study design incorporates the use of fictional scenarios, allowing participants to reflect on ethically charged situations while minimizing personal risk and encouraging openness. As these methodological safeguards are essential, they also introduce interpretative complexity: responses to hypothetical scenarios may not fully reflect real-life behaviors or decisions. Nevertheless, this approach represents a pragmatic balance between ethical sensitivity and the need for empirical insight in a challenging field of inquiry.

Given the limited data available, it remains unclear what specific content may be encountered in our context. As further precaution, participants are therefore provided with follow-up support conversations, if required to process any potentially distressing content discussed during the study interview in an appropriate and supportive manner. The value of such an approach for this purpose has been claimed on numerous occasions by ethical institutions and researchers worldwide, owing to the varying degrees of harm that distress in interview situations can cause when dealing with sensitive topics [19].

## Supplementary Information


Supplementary Material 1.


## Data Availability

No datasets were generated or analysed during the current study.
