# Supplementary material for: On the blind spot: ethical conflicts of health care professionals in addressing opioid diversion - a template study protocol for multinational qualitative research
Source: BMC Med Ethics. 2026 Aug 28;27:183. doi: 10.1186/s12910-026-01581-6 (PMC13523485; doi:10.1186/s12910-026-01581-6)
Supplement: Supplementary file 1 [file 12910_2026_1581_MOESM1_ESM.docx]

**Interview Guide**

[Read aloud by the interviewer]:

Thank you very much for taking the time to participate in our study. As you have already read in the study information, this interview will be audio-recorded and subsequently transcribed. All details that could reveal your identity will be removed.

Our interview focuses on opioid diversion, which refers to situations where opioid medications intended for patient care are taken or used by someone they were not prescribed for. In this interview, we will present a hypothetical scenario. It is important that you imagine this scenario taking place within your personal work context. However, the individuals involved remain fictional.

We will ask you several questions about how such a situation might unfold in your professional environment, how you might feel in such a situation, and what decisions you might make. Please note that there are no right or wrong answers. You are encouraged to share your thoughts freely.

If you have no questions, we will now begin the interview and start the recording.

Imagine the following situation occurs in your current work environment: There is a colleague in your team with whom you have been working for quite some time. This person is well-liked both in the clinic and within the team, and you also hold them in high regard. Lately, however, you have noticed some unusual behavior. For example, whenever opioids need to be administered, this colleague frequently volunteers to hand the medication directly to patients, even when this is not necessarily their responsibility. You may also notice that they tend to recommend or prescribe strong opioids or high dosages more readily when patients report pain. Or perhaps you observe that patients under this person's care often receive particularly high opioid doses. This leads you to wonder whether this could be a case of diversion.

| **Interview Question** | **Probing Prompts** |
| --- | --- |
| *(These questions will always be asked.)* | *(These questions will only be asked if necessary and are intended to maintain the flow of the conversation and to guide it purposefully.)* |
| 1. Please describe how such a situation could realistically unfold in your work environment. | How exactly could the situation unfold? Please feel free to describe it in more detail.  How did you imagine the person from the scenario?  Did you imagine a fellow [physician, etc.]? Or another role in your clinic? Why do you think you imagined that kind of person?  How exactly would you imagine diversion could take place in your field of work? |
| 2. What do you think could be the motive behind this person's diversion? | Do you have any other ideas about possible reasons?  Do you think these motives are justified or not? Why? |
| 3. What do you think about this situation and how would you feel in such a situation? | How does that feel exactly?  Do you perceive this situation as rather neutral, positive, or unpleasant? Why? |
| 4. What do you think about this person? | Do you have any further thoughts about this person?  Are there any differences in how you view this person as a colleague versus as an individual? |
| 4.1 To what extent could suspicion of diversion change your perception of this person in your working environment? | Do you notice any differences in your thoughts or attitudes toward this person?  What would change in your view of the person if an Opioid Use Disorder were the reason for the diversion? |
| 5. What moral aspects do you see as relevant in this conflict? | When we talk about morality, we mean your personal sense of what is right and wrong. It’s about the principles that guide how you decide whether an action is acceptable or not. |
| 6. What conflicts do you perceive in dealing with this situation? | Is there an inner conflict you could describe?  Would it make a difference if this person were your supervisor? If so, in what way?  What if this person were someone you supervise? Would that change anything?  Does it matter to you how close you are to the person personally? Why or why not? |
| 7. What would change in terms of the moral conflicts involved, depending on different motives for Diversion? | *[Depending on what was said before]*  What would change if the person had an opioid use disorder?  What would change if the opioids were sold for profit?  Would the extent of diversion influence your moral evaluation? For example, would it make a difference if the person diverted a prescription on a single occasion for a specific need, compared with repeatedly diverting over a longer period of time? Would you consider these situations ethically equivalent or different? |
| 8. What options for action do you see in this conflict situation? | Please describe in more detail what possible actions you see, and if possible, the advantages and disadvantages you associate with each.  Can you describe the protocols your hospital/clinic/department has in cases of suspected diversion? How do you feel about these protocols? Why? |
| 8.1 Which moral aspects could be considered for each possible solution – and which could not? | Please describe all your thoughts on this. |
| 8.1 How important are these moral aspects to you in this situation – and why? | Please feel free to create a personal hierarchy of importance. There are no right or wrong answers. Simply describe how you derive this hierarchy for yourself. |
| 9. How would you resolve the situation? | Which of the possible actions would you personally choose as a solution to the situation?  To what extent do you feel a personal responsibility to resolve the situation? Why do you feel that way? |
| 10. What would be your recommendation in this situation now? | How could this situation be handled in practice?  Please consider different levels, such as the structural level in healthcare, within the hospital, among professional groups, or on an individual level. |

Are there other ethical dilemmas you have encountered in your medical career? How do you compare these dilemmas to diversion [e.g., more/less complex, more/less concerning, etc.]

Are there any other aspects of this topic that are important to you but that we haven’t discussed yet?
